# International Differences, Challenges and Solutions in Wound Care

**DOI:** 10.1111/iwj.70173

**Published:** 2025-01-12

**Authors:** Keith Harding

**Affiliations:** ^1^ Cardiff University Cardiff UK

The *International Wound Journal* has a global readership and as such we must accept our responsibility to publish the best quality evidence that supports the use of new, old, natural or synthetic manufactured products. While the problems we face as a clinical community are for the most part common amongst us, the resources available may differ significantly in the clinical management of these patients. Evolution is a stepwise process and we are all at different stages within this specialisation of wound care.

Some recent examples of such differences have appeared in the *International Wound Journal* [1, 2, 3]. The manuscript from Uganda that is referenced [4] in this edition of the IWJ is a great example of a natural product that is talked about a great deal and is used in certain countries but has no or limited data to support its mode of action or where it is most beneficial to use it in practice. But when resources are limited, needs must.

As the clinical specialisation of our subject area continues its evolutionary journey, not all is equal in every corner of our globe. There is great potential for only believing new agents supported by commercial concerns are the only place to find effective wound therapeutics. Countries with limited resources would never be able to afford expensive products and as such we should keep an open mind on natural or folk remedies. This is not to provide a two‐tier approach to the selection of products but rather to enable some form of potentially beneficial care to be provided.

We still require evidence of effect and much more work is needed to evaluate dressings, devices, drugs, surgical approaches and biological agents as therapies for patients even in the most developed geographies of the world. We also require better study design, relevant outcome measures and improved diagnostic tests to ensure each study contributes new data to the wound community. In this perhaps we are all equal around the globe.

We must also accept that subject to sufficient proof of efficacy all interventions under test need evidence to support their use, even if naturally occurring, if we are to prevent losing potentially effective agents that have been used in the past but are seen as only natural products and are part of local folklore.
